# The Most Cited Papers in Osteoporosis and Related Research

**DOI:** 10.1155/2015/638934

**Published:** 2015-01-31

**Authors:** Lukas A. Holzer, Andreas Leithner, Gerold Holzer

**Affiliations:** ^1^Department of Orthopaedic Surgery, Medical University of Graz, 8036 Graz, Austria; ^2^Department of Orthopaedics, Medical University of Vienna, 1090 Vienna, Austria

## Abstract

Osteoporosis is a systemic disease of the bone that affects millions of people and causes burden for both the affected individual and health systems and societies worldwide. Since the 1970s much research has been done in the field of osteoporosis. The number of citations of a paper reflects its influence and importance to the field. Thomson ISI Web of Science database was searched to retrieve a list of the fifty most cited articles related to osteoporosis and its research. The fifty most cited articles in absolute numbers in the field of osteoporosis were cited from 877 to 3056 times (mean 1141 ± 537). Most papers were published in the basic science category (*n* = 23). 395 authors contributed; a single paper had between one and 62 authors (mean: 10.02 ± 9.9 authors). 12 authors (3.04%) contributed between 7 and 4 papers; 340 authors (86.1%) were at least named once. Corresponding authors were from eight countries with most contributions from the United States (*n* = 34, 68%). The majority of papers were published in the 1990s (*n* = 29). The list of 50 most cited papers presents citation classics in the field of osteoporosis and related research.

## 1. Introduction

Osteoporosis is a systemic disease of the bone that affects millions of people and causes burdens for both the affected individual and health systems and societies worldwide [1]. Osteoporosis is a multidisciplinary disease and therefore relevant to different medical specialities, for example, general medicine, internal medicine, endocrinology, gynaecology, orthopaedic surgery, and traumatology, but also to preclinical and basic disciplines such as physiology, pathology, and biomechanics.

Since the 1970s much research has been done in the field of osteoporosis. Research in osteoporosis poses a wide field and includes basic, clinical, and translational studies in the above mentioned specialities. Meanwhile several journals have been established that are dedicated to publishing articles related to the disease.

A citation is a quotation or a reference of published scientific work in books, book chapters, or articles [2]. The number of citations of published scientific work has been used as a marker to evaluate the level of its influence and importance. However, the number of citations may not be the only factor in determining the importance of scientific work in the field, but allows defining “citations classics” that could be used, for example, for educational purposes. Furthermore, the number of citations directly influences the impact factor of a journal, a generally accepted factor that determines its quality and importance [2].

Analyses of most cited papers have been performed in various medical specialties including anaesthesiology, gynaecology, urology, orthopaedic surgery, plastic surgery, or subspecialties such as pain management, critical care medicine, hand surgery, shoulder surgery, or orthopaedic joint replacement [3–12]. Furthermore, such lists exist for various pathologies or diseases such as pancreatitis, Parkinson's disease, depression, sepsis, or epilepsy [13–17]. However, no such study has been carried out in osteoporosis and related research.

The purpose of the present study is to determine scientific articles in the field of osteoporosis and related research that have been cited most frequently by other authors and to establish a ranking of the fifty most cited papers in the field by using the Thomson ISI Web of Science database.

## 2. Material and Methods

### 2.1. Search Strategy

In October 2013, Thomson ISI Web of Science database was searched for the following search terms: “osteoporosis,” “fracture,” “bone mineral density,” “bone density,” “bone mass,” “BMD,” “dual energy X-ray absorptiometry,” “DXA,” “DEXA,” “osteoclast,” “osteoblast,” “osteocyte,” “bone formation,” “bone resorption,” “hormone replacement therapy,” “estrogen replacement therapy,” “bisphosphonate,” “teriparatide,” “denosumab,” and “SERMs.”

All papers with the main focus of their research on osteoporosis and related basic, clinical and translational research were included in this study. Papers including the above used terms, but focusing on other research areas were excluded. The search output was then recorded and ranked according to the absolute number of highest citations. In cases with an identical absolute number of citations, the papers that had a higher citation density (see below) were ranked higher. A list of the fifty most cited articles was established.

Searches and analyses were done by individuals with a long-time experience in both basic and clinical research in osteoporosis.

### 2.2. Data Analyses

Each of the fifty most cited articles was reviewed and the following data was extracted: number of citations, authors, article title, journal title, publication year, and origin of corresponding author. Each paper was assigned to a single country in accordance with the corresponding author's address because the corresponding author is usually primarily and mainly responsible for the whole study project [18]. To evaluate the relative impact of a published paper, the citation density (“number of citations/years since publication”) was calculated as described before [12]. Furthermore, each article was analyzed and in case of a clinical study a level of evidence was attributed to the paper based on the guidelines for clinical articles by Oxford Centre for Evidence-Based Medicine 2011 Levels of Evidence (Oxford, UK: http://www.cebm.net/) [19]. Three categories were established: basic science, clinical science, and reviews and guidelines. The papers were analyzed and attributed to one of these categories.

## 3. Results

**Figure 1:**
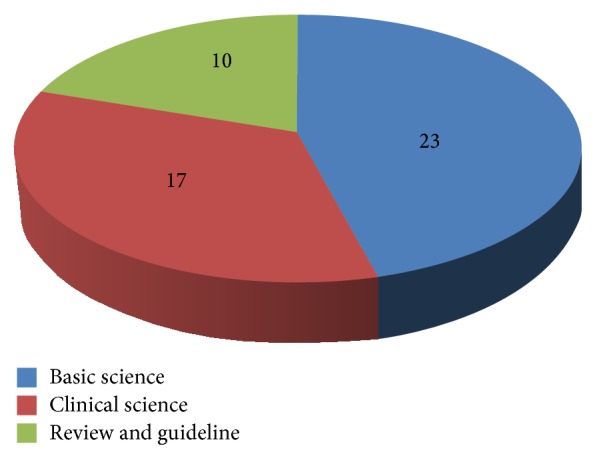
Distribution of categories.

**Figure 2:**
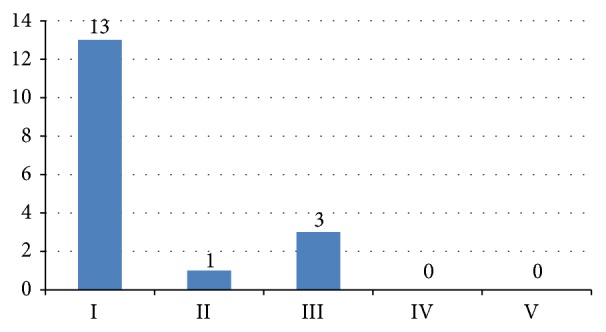
Level of evidence in the clinical papers.

The fifty most cited articles on osteoporosis and related research were cited from 877 to 3056 times (mean 1141 ± 537), the top ten papers at least 1741 times. For the whole list see Table 1. The most frequently cited paper was by Lacey et al. published in 1998 with a mean number of 184,5 citations per year. The top ten papers according to citation density can be seen in Table 2. The majority of articles could be attributed to the* basic science* category (*n* = 23). The distribution of the other categories can be seen in Figure 1. Level of evidence could be analyzed in 17 clinical papers (from the Clinical Science category) and can be seen in Table 1. The majority of papers (*n* = 13) were level of evidence I, one paper was level of evidence II, and three papers level of evidence III (see Figure 2).

Eleven search terms were found in the title of the papers; altogether these terms were found 70 times (mean: 6.4 ± 5.5 times). For the list of all search terms found see Table 3. Search terms “fracture” (*n* = 18) and “osteoporosis” (*n* = 14) were found most frequently (45.7% of all terms searched). Other terms searched were found between one and nine times (mean: 4.2 ± 2.9 times). More search terms were found in the abstract and the keywords of the papers.

All together 395 authors contributed to the papers of the top 50 list. A single paper had between one and 62 authors (mean: 10.02 ± 9.9 times). 12 authors (3.04%) contributed to between seven and four papers, 13 authors (3.3%) to three, and 30 twice (7.6%). 340 authors (86.1%) were named at least once. The top 12 authors are presented in Table 4.

**Figure 3:**
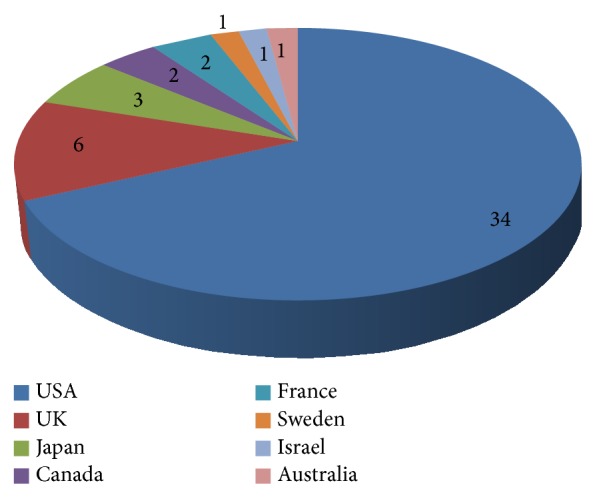
Distribution of countries.

Eight countries contributed to the Top 50 list (see Figure 3). Authors from the United States contributed most frequently as a corresponding author (*n* = 34, 68%), followed by authors from the United Kingdom (*n* = 6), Japan (*n* = 3), Canada and France (2 papers each), and Sweden, Israel, and Australia with one paper each.

**Figure 4:**
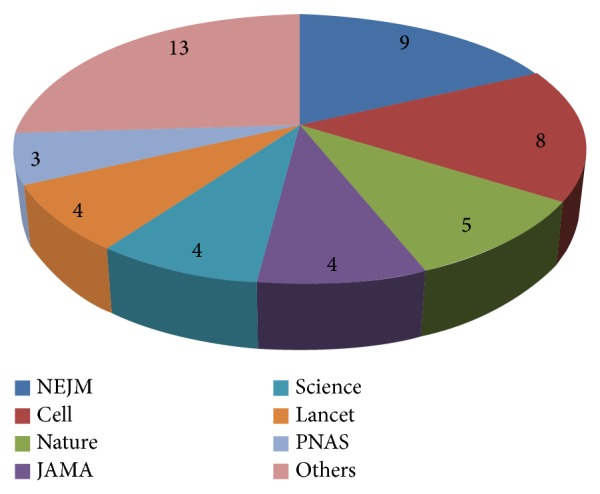
Distribution of journals.

Papers were published in 18 different journals publishing both basic and clinical research. Most papers (*n* = 9) were published in the New England Journal of Medicine. Other journals include Cell (*n* = 8),* Nature* (*n* = 5),* The Journal of the American Medical* Association, The Lancet, and Science (*n* = 4), Proceedings of the National Academy of Sciences (*n* = 3), Journal of Bone and Mineral Research and Journal of Clinical Investigation (two paper each), and American Journal of Clinical Nutrition, American Journal of Medicine, British Medical Journal, Cell and Tissue Research, Endocrinology, Endocrine Review, Epidemiology Reviews, Genes & Development, and Osteoporosis International one paper each. The distribution of the most cited papers in the various journals can be seen in Figure 4.

**Figure 5:**
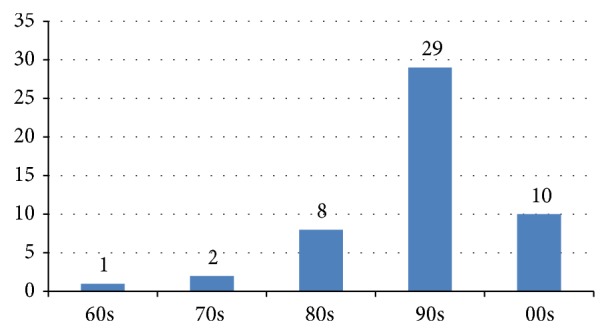
Number of papers published in each decade.

The number of most cited papers according to the decade of publication can be found in Figure 5. The majority of papers were published since 1990 (*n* = 39), whereas there is just one in the 1960s. Eight of the top 10 papers according to the citation density were published in the 1990s.

## 4. Discussion

In this study, Thomson ISI Web of Science was searched to analyze the most cited papers on osteoporosis and related research and to define a list of “citation classics” in this field.

In order to identify the most cited papers and cover all aspects and the whole field of osteoporosis and related research not only the term “osteoporosis” per se, but also processes involved in the biology and pathology of bone and bone metabolism as well as risk factors and the consequences of the disease were used. Furthermore, diagnostic and therapeutic options were considered. Altogether 20 search terms were used. The majority of articles could be attributed to “fractures” and “osteoporosis” (45.7% of papers altogether). This underlines the magnitude of osteoporosis and osteoporotic fractures. Eleven out of 20 search terms were found in the title of the papers, others in the abstract and keywords. All keywords were found, but some papers did not reach enough citations to be put onto the list of the Top 50.

Articles on osteoporosis and related research were cited up to 3056 times; the top ten papers according to absolute numbers were cited at least 1744 times. This fact shows the importance of osteoporosis as it affects a large number of patients worldwide and being of interest for many different medical specialities. Papers on other conditions such as acute pancreatitis had a maximum of 1281 citations [13]; the highest cited papers on septic conditions reached 2932 [14], whereas papers on epilepsy were cited even more frequently than osteoporosis (3749 citations) [15]. Top ranked articles in other fields such as papers on arthroscopic surgery or trauma reached about 500 citations [9, 10]. In orthopaedic paediatrics just four papers reached 100 citations [11].

The most cited paper in the present list was cited 3056 times. It is the oldest in the list and was published by a single author in 1956. The reason why this paper has been cited so often could be that “bone morphogenetic proteins” were described and for years their existence seemed to be the core of possible solutions for all aspects in bone metabolism [20].

395 authors contributed to the papers of the top 50 list. This reflects the wide range of osteoporosis and related research. Some authors contributed more than once; one author contributed seven times (Steven C. Cummings).

Regarding corresponding authors, in total, eight countries contributed to the list. Authors from the United States contributed most frequently as corresponding author (*n* = 34), followed by authors from the United Kingdom (*n* = 6), Japan (*n* = 3), Canada and France (2 papers each), and Sweden, Israel, and Australia with one paper each. All countries are highly industrialized and are ranked among the top in both economical and health-care expenditure. This is in accordance with previous analyses of other specialties or diseases [3–17].

The ranking is led by the United States with 34 papers (68%). This predominance is in accordance with other analyses where the US had the highest number of most cited papers [3–17]. This reflects the high frequency of research and the high scientific output in general and also in osteoporosis in the United States.

All papers were published in English. This clearly demonstrates the predominance of the English language in publications on osteoporosis and related research.

The spectrum of osteoporosis is also expressed in the large number of journals in which results of osteoporosis research are published. Papers were published in 18 different journals. Although there are journals focusing on osteoporosis itself and, more generally, on bone and bone metabolism, studies are also published in journals with a wide spectrum and more general medical background as such.

Papers were attributed to three different categories. Their distribution can be seen in Figure 1. Most papers were focusing on basic science.

Evidence Based Medicine has been introduced just recently using levels of evidence. Level of evidence could be analyzed in 17 clinical papers (from the Clinical Science category). According to the guidelines of the Oxford Centre for Evidence-Based Medicine the majority of articles (*n* = 13) were level of evidence I, one paper was level of evidence II, and three papers level of evidence III. This points to the fact that there is a high quality of study design and evidence in clinical osteoporosis research.

Interestingly, the majority of papers were published since 1990 (*n* = 39), whereas there is just one from the 1960s. Although bone changes were recognized quite early many significant advancements have been made in recent years. Considering this trend further developments in the field are to be expected.

This study has limitations. Identifying the 50 most cited papers they still remain a selection although using well defined criteria. So, important and influential papers with lower citation frequency might have been missed. In some cases the value of contribution to the field cannot be quantified by the number of citations. However, with respect to the aim of this study a bottom line is drawn. This approach seems to be more objective. Any other selection based on the importance of papers would depend on personal favour. The absolute or relative number of citations of articles can be influenced by several factors and does not necessarily reflect the importance of research that has been performed or has been published [12], nor does it directly translate into clinical practice changes. The search was performed in the Thomson ISI Web of Science database. Therefore citations of articles from other sources, such as textbooks, lectures, or digital media could not be considered. Another weakness might be the cross-sectional study design and research at a single point in time with focus on the absolute number of citations.

A list of 50 most cited papers in osteoporosis and related research covers a broad range of medical subspecialities. This is reflected by the number of journals and decades. Studies focusing on basic and clinical science of osteoporosis mainly dominate the literature in respect to absolute citations numbers. Considering the high number of high impact papers in the last two decades further developments in the field are to be expected.

## Figures and Tables

**Table 1:** The fifty citation classics in osteoporosis and related research.

Rank	Paper	Absolute number of citations	Citation density	Level of evidence
1	M. R. Urist, “Bone - formation by autoinduction,” *Science*, vol. 150, pp. 893–899, 1965.	3056	63,6	N/A

2	D. L. Lacey, E. Timms, H. L. Tan, M. J. Kelley, C. R. Dunstan, T. Burgess, R. Elliott, A. Colombero, G. Elliott, S. Scully, H. Hsu, J. Sullivan, N. Hawkins, E. Davy, C. Capparelli, A. Eli, Y. X. Qian, S. Kaufman, I. Sarosi, V. Shalhoub, G. Senaldi, J. Guo, J. Delaney, and W. J. Boyle, “Osteoprotegerin ligand is a cytokine that regulates osteoclast differentiation and activation,” *Cell*, vol. 93, pp. 165–176, 1998.	2768	184,5	N/A

3	J. M. Wozney, V. Rosen, A. J. Celeste, and L. M. Mitsock, “Novel regulators of bone-formation - molecular clones and activities,” *Science*, vol. 242, pp. 528–1534, 1988.	2662	106,4	N/A

4	W. S. Simonet, D. L. Lacey, C. R. Dunstan, M. Kelley, M. S. Chang, R. Luthy, H. Q. Nguyen, S. Wooden, L. Bennett, T. Boone, G. Shimamoto, M. DeRose, R. Elliott, A. Colombero, H. L. Tan, G. Trail, J. Sullivan, E. Davy, N. Bucay, L. Renshaw Gegg, T. M. Hughes, D. Hill, W. Pattison, P. Campbell, S. Sander, G. Van, J. Tarpley, P. Derby, R. Lee, and W. J. Boyle, *Cell*, vol. 89, pp. 309–319, 1997.	2629	164,3	N/A

5	H. Yasuda, N. Shima, N. Nakagawa, K. Yamaguchi, M. Kinosaki, S. Mochizuki, A. Tomoyasu, K. Yano, M. Goto, A. Murakami, E. Tsuda, T. Morinaga, K. Higashio, N. Udagawa, N. Takahashi, and T. Suda, “Osteoclast differentiation factor is a ligand for osteoprotegerin osteoclastogenesis-inhibitory factor and is identical to TRANCE/RANKL,” *Proceedings of the National Academy of Sciences of the United States of America*, vol. 95, pp. 3597–3602, 1998.	2206	147	N/A

6	T. Komori, H. Yagi, S. Nomura, A. Yamaguchi, K. Sasaki, K. Sasaki, K. Deguchi, Y. Shimizu, R. T. Bronson, Y. H. Gao, M. Inada, M. Sato, R. Okamoto, Y. Kitamura, S. Yoshiki, and T. Kishimoto, “Targeted disruption of Cbfa1 results in a complete lack of bone formation owing to aturational arrest of osteoblasts,” *Cell*, vol. 89, pp. 755–764, 1998.	2132	133,2	N/A

7	S. R. Cumming, M. C. Nevitt, W. S. Browner, K. Stone, K. M. Fox, K. E. Ensrud, J. C. Cauley, D. Black, and T. M. Vogt, “Risk-factors for hip fracture in white women,” *The New England Journal of Medicine*, vol. 332, pp. 767–773, 1995.	2102	116,7	I

8	D. M. Black, S. R. Cumming, D.B. Karpf, J. A. Cauley, D. E. Thompson, M. C. Nevitt, D. C. Bauer, H. K. Genant, W. L. Haskell, R. Marcus, S. M. Ott, J. C. Torner, S. A. Quandt, T. F. Reiss, and K. E. Ensrud, “Randomised trial of effect of alendronate on risk of fracture in women with existing vertebral fractures,” *Lancet*, vol. 348, pp. 1535–1541, 1996.	2067	121,5	I

9	M. C. Chapuy, M. E. Arlot, F. Duboeuf, J. Brun, B. Crouzet, S. Arnaud, P. D. Delmas, and P. J. Meunier, “Vitamin-D(3) and calcium to prevent hip-fractures in elderly women,” *The New England Journal of Medicine*, vol. 23, pp. 1637–1642, 1992.	1771	84,3	I

10	Y. Y. Kong, H. Yoshida, I. Sarosi, H. L. Tan, E. Timms, C. Capparelli, S. Morony, A. J. Oliveira-dos-Santos, G. Van, A. Itie, W. Khoo, A. Wakeham, C. R. Dunstan, D. L. Lacey, T. W. Mak, W. J. Boyle, and J. M. Penninger, “OPGL is a key regulator of osteoclastogenesis, lymphocyte development and lymph-node organogenesis,” *Nature*, vol. 397, pp. 315–323, 1999.	1741	124,3	N/A

11	B. Ettinger, D. M. Black, B. H. Mitlak, R. K. Knickerbocker, T. Nickelsen, H. K. Genant, C. Christiansen, P. D. Delmas, J. R. Zanchetta, J. Stakkestad, C. C. Gluer, K. Krueger, F. J. Cohen, S. Eckert, K. E. Ensrud, L. V. Avioli, P. Lips, and S. R. Cummings, “Reduction of vertebral fracture risk in postmenopausal women with osteoporosis treated with raloxifene,” *The Journal of the American Medical Association*, vol. 282, pp. 637–645, 1999.	1732	123,7	I

12	B. L. Riggs, and L. J. Melton, “Involutional Osteoporosis,” *The New England Journal of Medicine*, vol. 314, pp. 1676–1686, 1986.	1719	63,6	N/A

13	D. Marshall, O. Johnell, and H. Wedel, “Meta-analysis of how well measures of bone mineral density predict occurrence of osteoporotic fractures,” *British Medical Journal*, vol. 312, pp. 1254–1259, 1996.	1689	99,3	I

14	R. M. Neer, C. D. Arnaud, J. R. Zanchetta, R. Prince, G. A. Gaich, J. Y. Reginster, A. B. Hodsman, E. F. Eriksen, S. Ish-Shalom, H. K. Genant, O. H. Wang, and B. H. Mitlak, “Effect of parathyroid hormone (1-34) on fractures and bone mineral density in postmenopausal women with osteoporosis,” *Lancet*, vol. 344, pp. 1434–1441, 2001.	1686	140,5	I

15	S. R. Cummings, D. M. Black, M. C. Nevitt, W. Browner, J. Cauley, K. Ensrud, H. K. Genant, L. Palermo, J. Scott, and T. M. Vogt, “Bone-density at various sites for prediction of hip-fractures,” *The New England Journal of Medicine*, vol. 341, pp. 72–75, 1993.	1667	83,3	I

16	W. J. Boyle, W. S. Simonet, and D. L. Lacey, “Osteoclast differentiation and activation,” *Cell*, vol. 423, pp. 337–342, 2003.	1651	165,1	N/A

17	P. Soriano, C. Montgomery, R. Geske, and A. Bradley, “Targeted disruption of the c-src protooncogene leads to osteopetrosis in mice,” *Nature*, vol. 64, pp. 693–702, 1991.	1609	73,1	N/A

18	J. A. Kanis, L. J. Melton, C. Christiansen, C. C. Johnston, and N. Khaltaev, “Perspective-the diagnosis of osteoporosis,” *Cell*, vol. 9, pp. 1137–1141, 1994.	1532	80,6	N/A

19	F. Otto, A. P. Thornell, T. Crompton, A. Denzel, K. C. Gilmour, I. R. Rosewell, G. W. H. Stamp, R. S. P. Beddington, S. Mundlos, B. R. Olsen, P. B. Selby, and M. J. Owen, “Cbfa1, a candidate gene for cleidocranial dysplasia syndrome, is essential for osteoblast differentiation and bone development,” *Journal of Bone and Mineral Research*, vol. 89, pp. 765–771, 1997.	1529	95,5	N/A

20	U. A. Liberman, S. R. Weiss, J. Broll, H. W. Minne, H. Quan, N. H. Bell, J. Rodriguezportales, R. W. Downs, J. Dequeker, M. Favus, E. Seeman, R. R. Recker, T. Capizzi, A. C. Santora, A. Lombardi, R. V. Shah, L. J. Hirsch, and D. B. Karpf, “Effect of oral alendronate on bone-mineral density and the incidence of fractures in postmenopausal osteoporosis,” *The New England Journal of Medicine*, vol. 333, pp. 1437–1443, 1995.	1484	82,4	I

21	N. A. Morrison, J. C. Qi, A. Tokita, P. J. Kelly, L. Crofts, T. V. Nguyen, P. N. Sambrook, and J. A. Eisman, “Prediction of bone-density from vitamin-d receptor alleles,” *Nature*, vol. 367, pp. 284–287, 1994.	1372	72,2	N/A

22	S. L. Teitelbaum, “Bone resorption by osteoclasts,” *Science*, vol. 289, pp. 1504–1508, 2000.	1370	105,3	N/A

23	N. Bucay, I. Sarosi, C. R. Dunstan, S. Morony, J. Tarpley, C. Capparelli, S. Scully, H. L. Tan, W. L. Xu, D. L. Lacey, W. J. Boyle, and W. S. Simonet, “Osteoprotegerin-deficient mice develop early onset osteoporosis and arterial calcification,” *Genes & Development*, vol. 12, pp. 1260–1268, 1998.	1292	86,1	N/A

24	S. T. Harris, N. B. Watts, H. K. Genant, C. D. McKeever, T. Hangartner, M. Keller, C. H. Chesnut, J. Brown, E. F. Eriksen, M. S. Hoseyni, D. W. Axelrod, and P. D. Miller, “Effects of risedronate treatment on vertebral and nonvertebral fractures in women with postmenopausal osteoporosis - a randomized controlled trial,” *The Journal of the American Medical Association*, vol. 282, pp. 1344–1352, 1999.	1291	92,2	I

25	S. R. Cummings, J. L. Kelsey, M. C. Nevitt, and K. J. Odowd, “Epidemiology of osteoporosis and osteoporotic fractures,” *Epidemiologic Reviews*, vol. 7, pp. 178–208, 1985.	1259	44,9	N/A

26	A. Klibanski, L. Adams-Campbell, T. Bassford, S. N. Blair, S. D. Boden, K. Dickersin, D. R. Gifford, L. Glasse, S. R. Goldring, K. Hruska, S. R. Johnson, L. K. McCauley, and W. E. Russell, “Osteoporosis prevention, diagnosis, and therapy,” *The Journal of the American Medical Association*, vol. 285, pp. 785–795, 2001.	1254	104,5	N/A

27	S. R. Cummings, D. M. Black, D. E. Thompson, W. B. Applegate, E. Barrett-Connor, T. A. Musliner, L. Palermo, R. Prineas, S. M. Rubin, J. C. Scott, T. Vogt, R. Wallace, A. J. Yates, and A. Z. LaCroix, “Effect of alendronate on risk of fracture in women with low bone density but without vertebral fractures - Results from the fracture intervention trial,” *The Journal of the American Medical Association*, vol. 280, pp. 2077–2082, 1998.	1245	83	I

28	D. R. Bertolini, G. E. Nedwin, and T. S. Bringman, “Stimulation of bone-resorption and inhibition of bone-formation invitro by human-tumor necrosis factors,” *Nature*, vol. 319, pp. 516–518, 1986.	1176	43,5	N/A

29	B. DawsonHughes, S. S. Harris, E. A. Krall, and G. E. Dallal, “Effect of calcium and vitamin D supplementation on bone, density in men and women 65 years of age or older,” *The New England Journal of Medicine*, vol. 337, pp. 670–676, 1997.	1170	73,1	I

30	P. D. Delmas, N. H. Bjarnason, B. H. Mitlak, A. C. Ravoux, A. S. Shah, W. J. Huster, M. Draper, and C. Christiansen, “Effects of raloxifene on bone mineral density, serum cholesterol concentrations, and uterine endometrium in postmenopausal women*,” The New England Journal of Medicine*, vol. 337, pp. 1641–1647, 1997.	1164	72,7	I

31	T. Suda, N. Takahashi, N. Udagawa, E. Jimi, M. T. Gillespie, T. J. Martin, “Modulation of osteoclast differentiation and function by the new members of the tumor necrosis factor receptor and ligand families,” *Endocrine Reviews*, vol. 20, pp. 345–357, 1999.	1162	83	N/A

32	K. Nakashima, X. Zhou, G. Kunkel, Z. P. Zhang, J. M. Deng, R. R. Behringer, and B. de Crombrugghe, “The novel zinc finger-containing transcription factor Osterix is required for osteoblast differentiation and bone formation,” *Cell*, vol. 108, pp. 17–29, 2002.	1143	103,9	N/A

33	A. M. Parfitt, C. H. E. Mathews, A. R. Villanueva, M. Kleerekoper, B. Frame, and D.S. Rao, “ Relationships between surface, volume, and thickness of iliac trabecular bone in aging and in osteoporosis-implications for the microanatomic and cellular mechanisms of bone loss,” *The Journal of Clinical Investigation*, vol. 72, pp. 1396–1409, 1983.	1117	37,2	N/A

34	S. R. Cummings, and L. J. Melton, “Epidemiology and outcomes of osteoporotic fractures,” *Proceedings of the National Academy of Sciences of the United States of America*, vol. 359, pp. 1761–1767, 2002.	1089	99	N/A

35	E. A. Wang, V. Rosen, J. S. Dalessandro, M. Bauduy, P. Cordes, T. Harada, D. I. Israel, R. M. Hewick, K. M. Kerns, P. Lapan, D. P. Luxenberg, D. McQuaid, I. K. Moutsatsos, J. Nove, and J. M. Wozney, “Recombinant human bone morphogenetic protein induces bone-formation,” *Lancet*, vol. 87, pp. 2220–2224, 1990.	1077	46,8	N/A

36	S. C. Manolagas, and R. L. Jilka, “Mechanisms of disease - bone-marrow, cytokines, and bone remodeling - emerging insights into the pathophysiology of osteoporosis,” *The New England Journal of Medicine*,vol. 332, pp. 305–311, 1995.	1047	58,1	N/A

37	C. Cooper, G. Campion, and L. J. Melton, “Hip-fractures in the elderly - a worldwide projection,” *Osteoporosis International*,vol. 2, pp. 285–289, 1992.	1037	49,3	N/A

38	H. K. Genant, C. Y. Wu, C. Vankuijk, and M. C. Nevitt, “Vertebral fracture assessment using a semiquantitative technique,” *Science*, vol. 8, pp. 1137–1148, 1993.	1018	50,9	III

39	Y. Q. Gong, R. B. Slee, N. Fukai, Y. Q. Gong, R. B. Slee, N. Fukai, G. Rawadi, S. Roman-Roman, A. M. Reginato, H. W. Wang, T. Cundy, F. H. Glorieux, D. Lev, M. Zacharin, K. Oexle, J. Marcelino, W. Suwairi, S. Heeger, G. Sabatakos, S. Apte, W. N. Adkins, J. Allgrove, M. Arslan-Kirchner, J. A. Batch, P. Beighton, G. C. M. Black, R. G. Boles, L. M. Boon, C. Borrone, H. G. Brunner, G. F. Carle, B. Dallapiccola, A. De Paepe, B. Floege, M. L. Halfhide, B. Hall, R. C. Hennekam, T. Hirose, A. Jans, H. Juppner, C. A. Kim, K. Keppler-Noreuil, A. Kohlschuetter, D. LaCombe, M. Lambert, E. Lemyre, T. Letteboer, L. Peltonen, R. S. Ramesar, M. Romanengo, H. Somer, E. Steichen-Gersdorf, B. Steinmann, B. Sullivan, A. Superti-Furga, W. Swoboda, M. J. van den Boogaard, W. Van Hul, M. Vikkula, M. Votruba, B. Zabel, T. Garcia, R. Baron, B. R. Olsen, and M. L. Warman, “LDL receptor-related protein 5 (LRP5) affects bone accrual and eye development,” *Cell*, vol. 107, pp. 513–523, 2001.	1009	84	N/A

40	R. L. Jilka, G. Hangoc, G. Girasole, G. Passeri, D. C. Williams, J. S. Abrams, B. Boyce, H. Broxmeyer, and S. C. Manolagas, “Increased osteoclast development after estrogen loss - mediation by interleukin-6,” *The American Journal of Clinical Nutrition*, vol. 257, pp. 88–91, 1992.	1008	48	N/A

41	R. B. Mazess, H. S. Barden, J. P. Bisek, and J. Hanson, “Dual-energy X-ray absorptiometry for total-body and regional bone-mineral and soft-tissue composition,” *Journal of Bone and Mineral Research*, vol. 51, pp. 1106–1112, 1990.	1000	43,4	III

42	B. L. Riggs, H. W. Wahner, W. L. Dunn, R. B. Mazess, K. P. Offord, and L. J. Melton, “Differential changes in bone-mineral density of the appendicular and axial skeleton with aging - relationship to spinal osteoporosis,” *The Journal of Clinical Investigation*, vol. 67, pp. 328–335, 1981.	971	30,3	III

43	C. Maniatopoulos, J. Sodek, and A. H. Melcher, “Bone-formation invitro by stromal cells obtained from bone-marrow of young-adult rats,” *Cell and Tissue Research*, vol. 254, pp. 317–330, 1988.	964	38,5	N/A

44	M. R. McClung, P. Geusens, P. D. Miller, H. Zippel, W. G. Bensen, C. Roux, S. Adami, I. Fogelman, T. Diamond, R. Eastell, P. J. Meunier, J. Y. Reginster, R. D. Wasnich, M. Greenwald, J. Kaufman, and C. H. Chestnut, “Effect of risedronate on the risk of hip fracture in elderly women,” *The New England Journal of Medicine*, vol. 344, pp. 333–340, 2001.	954	79,5	I

45	P. Ducy, M. Amling, S. Takeda, M. Priemel, A. F. Schilling, F. T. Beil, J. H. Shen, C. Vinson, J. M. Rueger, and G. Karsenty, “Leptin inhibits bone formation through a hypothalamic relay: A central control of bone mass,” *Cell*, vol. 100, pp. 197–207, 2000.	946	72,7	N/A

46	R. Lindsay, D. M. Hart, J. M. Aitken, E. B. MacDonald, J. B. Anderson, and A. C. Clarke, “Long-term prevention of postmenopausal osteoporosis by estrogen - evidence for an increased bone mass after delayed onset of estrogen-treatment*,” Lancet*, vol. 1, pp. 1038–1041, 1976.	938	25,3	II

47	H. L. Hsu, D. L. Lacey, C. R. Dunstan, I. Solovyev, A. Colombero, E. Timms, H. L. Tan, G. Elliott, M. J. Kelley, I. Sarosi, L. Wang, X. Z. Xia, R. Elliott, L. Chiu, T. Black, S. Scully, C. Capparelli, S. Morony, G. Shimamoto, M. B. Bass, and W. J. Boyle, “Tumor necrosis factor receptor family member RANK mediates osteoclast differentiation and activation induced by osteoprotegerin ligand,” *Nature*, vol. 96, pp. 3540–3545, 1999.	904	64,5	N/A

48	M. Gowen, D. D. Wood, E. J. Ihrie, M. K. B. McGuire, and R. G. G. Russell, “An interleukin-1 like factor stimulates bone-resorption in vitro,” *Proceedings of the National Academy of Sciences of the United States of America*, vol. 306, pp. 378–380, 1983.	903	30,1	N/A

49	D. C. Klein, and L. G. Raisz, “Prostaglandins - stimulation of bone resorption in tissue culture,” *Endocrinology*, vol. 86, pp. 1436–40, 1970.	880	20,4	N/A

50	S. C. Manolagas, “Birth and death of bone cells: Basic regulatory mechanisms and implications for the pathogenesis and treatment of osteoporosis,” *American Journal of Medicine*, vol. 21, pp. 115–137, 2000.	877	67,4	N/A

**Table 2:** The top ten papers according to citation density.

Rank	Paper	Absolute number of citations	Citation density	Level of evidence
1	D. L. Lacey, E. Timms, and H. L. Tan, M. J. Kelley, C. R. Dunstan, T. Burgess, R. Elliott, A. Colombero, G. Elliott, S. Scully, H. Hsu, J. Sullivan, N. Hawkins, E. Davy, C. Capparelli, A. Eli, Y. X. Qian, S. Kaufman, I. Sarosi, V. Shalhoub, G. Senaldi, J. Guo, J. Delaney, and W. J. Boyle, “Osteoprotegerin ligand is a cytokine that regulates osteoclast differentiation and activation,” *Cell*, vol. 93, pp. 165–176, 1998.	2768	184,5	N/A

2	W. J. Boyle, W. S. Simonet, and D. L. Lacey, “Osteoclast differentiation and activation,” *Cell*, vol. 423, pp. 337–342, 2003.	1651	165,1	N/A

3	W. S. Simonet, D. L. Lacey, C. R. Dunstan, M. Kelley, M. S. Chang, R. Luthy, H. Q. Nguyen, S. Wooden, L. Bennett, T. Boone, G. Shimamoto, M. DeRose, R. Elliott, A. Colombero, H. L. Tan, G. Trail, J. Sullivan, E. Davy, N. Bucay, L. Renshaw Gegg, T. M. Hughes, D. Hill, W. Pattison, P. Campbell, S. Sander, G. Van, J. Tarpley, P. Derby, R. Lee, and W. J. Boyle, *Cell*, vol. 89, pp. 309–319, 1997.	2629	164,3	N/A

4	H. Yasuda, N. Shima, N. Nakagawa, K. Yamaguchi, M. Kinosaki, S. Mochizuki, A. Tomoyasu, K. Yano, M. Goto, A. Murakami, E. Tsuda, T. Morinaga, K. Higashio, N. Udagawa, N. Takahashi, and T. Suda, “Osteoclast differentiation factor is a ligand for osteoprotegerin osteoclastogenesis-inhibitory factor and is identical to TRANCE/RANKL,” *Proceedings of the National Academy of Sciences of the United States of America*, vol. 95, pp. 3597–3602, 1998.	2206	147	N/A

5	T. Komori, H. Yagi, S. Nomura, A. Yamaguchi, K. Sasaki, K. Sasaki, K. Deguchi, Y. Shimizu, R. T. Bronson, Y. H. Gao, M. Inada, M. Sato, R. Okamoto, Y. Kitamura, S. Yoshiki, and T. Kishimoto, “Targeted disruption of Cbfa1 results in a complete lack of bone formation owing to aturational arrest of osteoblasts,” *Cell*, vol. 89, pp. 755–764, 1998.	2132	133,2	N/A

6	Y. Y. Kong, H. Yoshida, I. Sarosi, H. L. Tan, E. Timms, C. Capparelli, S. Morony, A. J. Oliveira-dos-Santos, G. Van, A. Itie, W. Khoo, A. Wakeham, C. R. Dunstan, D. L. Lacey, T. W. Mak, W. J. Boyle, and J. M. Penninger, “OPGL is a key regulator of osteoclastogenesis, lymphocyte development and lymph-node organogenesis,” *Nature*, vol. 397, pp. 315–323, 1999.	1741	124,3	N/A

7	B. Ettinger, D. M. Black, B. H. Mitlak, R. K. Knickerbocker, T. Nickelsen, H. K. Genant, C. Christiansen, P. D. Delmas, J. R. Zanchetta, J. Stakkestad, C. C. Gluer, K. Krueger, F. J. Cohen, S. Eckert, K. E. Ensrud, L. V. Avioli, P. Lips, and S. R. Cummings, “Reduction of vertebral fracture risk in postmenopausal women with osteoporosis treated with raloxifene,” *The Journal of the American Medical Association*, vol. 282, pp. 637–645, 1999.	1732	123,7	I

8	D. M. Black, S. R. Cummings, D. B. Karpf, J. A. Cauley, D. E. Thompson, M. C. Nevitt, D. C. Bauer, H. K. Genant, W. L. Haskell, R. Marcus, S. M. Ott, J. C. Torner, S. A. Quandt, T. F. Reiss, and K. E. Ensrud, “Randomised trial of effect of alendronate on risk of fracture in women with existing vertebral fractures,” *Lancet*, vol. 348, pp. 1535–1541, 1996.	2067	121,5	I

9	S. R. Cummings, M. C. Nevitt, W. S. Browner, K. Stone, K. M. Fox, K. E. Ensrud, J. C. Cauley, D. Black, and T. M. Vogt, “Risk-factors for hip fracture in white women,” *The New England Journal of Medicine*, vol. 332, pp. 767–773, 1995.	2102	116,7	I

10	J. M. Wozney, V. Rosen, A. J. Celeste, and L. M. Mitsock, “Novel regulators of bone-formation - molecular clones and activities,” *Science*, vol. 242, pp. 528–1534, 1988.	2662	106,4	N/A

**Table 3:** Most frequent search terms (*n* = 11).

Search term	Number of search terms found
Fracture	18
Osteoporosis	14
Bone formation	9
Osteoclast	8
Bone mineral density	5
Bone density	5
Bone resorption	4
Osteoblast	3
Bone mass	2
Dual energy X-ray absorptiometry	1
Estrogen replacement therapy	1

**Table 4:** Top 12 authors who contributed to the top 50 list.

Author's name	Number of papers by author
S. R. Cummings	7
W. J. Boyle	6
H. K. Gennant	6
D. L. Lacey	6
D. M. Black	5
C. R. Dunstan	5
L. J. Melton	5
M. C. Nevitt	5
H. L. Tan	5
C. Caparelli	4
K. E. Ensrud	4
I. Sarosi	4
